# Choc anaphylactique au cours de la chirurgie de kyste hydatique du foie: à propos d’un cas

**Published:** 2010-08-07

**Authors:** Qamouss Youssef, Seddiqui Rachid, Aissawi Younes, Filali Karim, Boughalem Mohamed

**Affiliations:** 1 Service de Réanimation, Hôpital Militaire Avicenne Marrakech, Maroc

**Keywords:** Choc anaphylactique, kyste hydatique, Maroc

## Abstract

Nous rapportons l’observation d’un choc anaphylactique survenue au cours d’une chirurgie de kystes hydatiques hépatiques multiples. Il s’agit d’un enfant de 13 ans, de sexe féminin, sans antécédents pathologiques notables, en particulier allergique, programmé pour cure chirurgicale de kystes hydatiques hépatiques multiples. L’examen préopératoire est strictement normal. La patiente est classée ASA (American Society of Anaesthesiologists) I. L’intervention est réalisée sous anesthésie générale. Au moment de la manipulation du deuxième kyste, une augmentation des pressions d’insufflation apparaît, avec des râles sibilants au niveau des deux champs pulmonaires et une désaturation, suivi d’un collapsus vasculaire avec hypotension artérielle 50/30. La prise en charge est débutée aussitôt par un remplissage vasculaire par du sérum salé 9%, suivi par des bolus d’adrénaline de 0,1mg à trois reprises, puis d’une perfusion continue à raison. L’adrénaline est arrêtée au bout de 48 heures et la patiente est adressée au service de chirurgie à j+4 de l’intervention. Le choc anaphylactique est une complication connue de la rupture intra péritonéale spontanée ou post-traumatique du kyste hydatique du foie. Sa survenue en peropératoire est devenue exceptionnelle, grâce aux mesures de prévention chirurgicale entretenues en peropératoire par l’éviction de manipulation du kyste, de le vider avant d’injecter le scolicide, et de ne pas l’administrer sous forte pression.

## Introduction

Le choc anaphylactique au cours de la chirurgie des kystes hydatiques, est une complication potentiellement grave, imposant un diagnostic rapide et un traitement adapté. Toutefois, les accidents peropératoires se caractérisent par leur brutalité de survenue, leur gravité, ainsi que la difficulté du diagnostic étiologique du collapsus hémodynamique. Ce collapsus peut être d’origine anaphylactique, hémorragique ou toxique [1,2].

Nous rapportons l’observation d’un choc anaphylactique survenant au cours d’une chirurgie de kystes hydatiques hépatiques multiples.

## Patient et observation

Il s’agit d’un enfant de 13 ans, de sexe féminin, sans antécédents pathologiques notables, en particulier allergiques, programmé pour cure chirurgicale de kystes hydatiques hépatiques multiples. L’examen préopératoire est strictement normal, en dehors d’une hépatomégalie. L’analyse des données biologiques (numération formule sanguine (NFS), ionogramme sanguin, hémostase et bilan hépatique) n’a pas montré de particularité. La sérologie hydatique est positive. La radiographie pulmonaire est normale. La patiente est classée ASA I (selon la classification de l’American Society of Anaesthesiology qui précise le risque chirurgical en fonction du terrain), la prémédication a été assuré par de l’hydroxyzine 2 mg/kg.

L’intervention est réalisée sous anesthésie générale par du midazolam (0,2mg/kg), fentanyl (2,5µg/kg) et du vécuronium (0,1mg/kg). L’anesthésie est entretenue par de l’halothane et du fentanyl, le peropératoire s’est déroulé sans incidents.

Jusqu’au moment de la manipulation du deuxième kyste, une nette augmentation des pressions d’insufflation apparaît, avec des râles sibilants au niveau des deux champs pulmonaires et une désaturation, suivi d’un collapsus vasculaire avec hypotension artérielle 50/30. Aucune manifestation cutanée n’a été observée. La prise en charge est débutée aussitôt par un remplissage vasculaire par du sérum salé (SS) 0,9% (25cc/kg), à deux reprises et la prise d’une voie veineuse centrale, et l’administration intraveineuse d’un bolus d’adrénaline de 0,1mg à trois reprises, suivie d’une perfusion continue à raison de 0,5µg/kg/mn, devant la persistance du collapsus vasculaire.

L’adrénaline est arrêtée progressivement au bout de 48 heures et la patiente est adressée au service de chirurgie après quatre jours de séjour en unité de soins intensifs et après sevrage en adrénaline et après stabilité cardio-vasculaire et pleuropulmonaire.

## Discussion

Le choc anaphylactique par rupture de kyste hydatique a été décrit la première fois en 1909 par M. Chauffard [3]. En 1935, M. Lemaire insistait sur le rôle de la fraction protéique non diffusible du liquide hydatique, constituant pour lui un antigène (Ag) puissant.

Les fuites ou les ruptures accidentelles peropératoires des kystes hydatiques du foie peuvent être à l’origine de réactions anaphylactiques secondaires à une absorption systémique du liquide hydatique [3,4]. Le mécanisme de ces réactions est complexe. Dans certains cas, il s’agit typiquement d’une réaction d’hypersensibilité de type I liée à des immunoglobulines E en réponse à la forte concentration plasmatique des Ag d’Ecchinococcus. La réaction anaphylactique ou anaphylactoïde peut être aussi secondaire à une activation du complément avec libération d’anaphylatoxines. In vitro, le liquide hydatique active la voie alterne du complément avec formation de C3a. Les manifestations anaphylactiques peuvent revêtir des aspects très différents à type de bronchospasme [3] et/ou de choc anaphylactique. Dans notre cas, c’était la forme complète, avec absence des signes cutanés. Les observations de Terpstra et celles de Jakubowski ont le mérite d’insister sur la rareté de ce type d’accident, sur l’absence de signes prémonitoires, et sur la difficulté du diagnostic devant un collapsus hémodynamique brutal après la stérilisation et l’évacuation du kyste [5].

Le choc anaphylactique doit être évoqué devant l’association à des degrés variables de signes cutanés, d’un bronchospasme ou d’un collapsus inexpliqué par un saignement peropératoires, et sa survenue à distance de l’induction.

Les accidents cardiovasculaires aigus observés en peropératoire ne revêtent pas toujours le caractère de choc anaphylactique. Une origine toxique est alors invoquée, le formol étant le produit scolicide le plus souvent mis en cause dans ces complications [5,6]. La toxicité d’un passage sanguin en quantité importante de formol est certaine et un arrêt cardiaque lié à une utilisation par erreur d’une solution de formol à 35 % a d’ailleurs été rapporté [7]. De même lorsque le cétrimide passe dans le sang, il peut être responsable d’accidents aigus allant jusqu’à l’arrêt cardiaque.

Dans notre cas, on a procédé à une ponction-aspiration du liquide hydatique sous protection par des champs. Les manifestations anaphylactoïdes sont survenues de façon typique associant des signes respiratoires et cardio-vasculaires.

Les patients présentant une réaction anaphylactoïde bénéficieront d’un bilan biologique, celui-ci comprend la mesure des taux circulants de tryptase sérique et d’histamine plasmatique destinée à confirmer la réalité du choc anaphylactique, et la recherche d’immunoglobulines E spécifiques. Les prélèvements sanguins sont réalisés sur un tube sec (7 mL) et un tube EDTA (7 mL) dès que la situation clinique est maîtrisée. Le dosage d’histamine doit être réalisé précocement dans l’heure qui suit le début des signes, celui de tryptase idéalement environ une heure après le début des signes. Les tubes doivent être transmis au laboratoire dans les deux heures. En cas d’impossibilité, ils peuvent être conservés dans le réfrigérateur à + 4^o^ C pendant 12 heures au maximum.

La sévérité des manifestations cliniques, et l’efficacité des mesures thérapeutiques peuvent varier de manière très importante d’une situation à l’autre. De plus, en l’absence d’étude clinique contrôlée, les recommandations thérapeutiques font habituellement appel à des avis d’experts. En conséquence, la pertinence des choix thérapeutiques lors de la survenue d’une réaction anaphylactoïde repose sur le jugement du clinicien qui doit tenir compte des manifestations cliniques et des options diagnostiques et thérapeutiques disponibles. Le traitement de l’anaphylaxie a pour objectif d’interrompre l’exposition du sujet à l’allergène incriminé, de minimiser les effets induits par les médiateurs libérés et d’en inhiber la production et la libération. Le traitement doit être institué dans les meilleurs délais et repose sur des principes consensuels [7].

La procédure opératoire chirurgicale est interrompue chaque fois que possible. Le contrôle de la liberté des voies aériennes est impératif. Le recours à une administration d’oxygène pure doit être systématique. La mise en place d’un accès veineux permettant une perfusion à débit élevé et le monitorage de l’électrocardiogramme et de la pression artérielle doivent être institués. Le patient doit être allongé et les membres supérieurs surélevés. Ces mesures doivent être appliquées dans tous les cas.

Un remplissage vasculaire rapide doit être associé à la prescription d’amine vasopressive. Le remplissage doit être institué sans délai, pendant la préparation de l’adrénaline. Il consiste en la perfusion rapide de cristalloïdes (10 à 25 mL·kg^-1^) en 20 minutes, répétée si besoin.

L’adrénaline est le médicament de choix. En première intention, associée au remplissage vasculaire, l’adrénaline s’oppose aux effets délétères des médiateurs libérés au cours de la réaction anaphylactoïde par ses propriétés vasoconstrictrices (agoniste alpha 1), inotrope positive (agoniste beta 1) et bronchodilatatrice (agoniste beta 2). Elle permet également de diminuer la libération des médiateurs par les mastocytes et les basophiles. On injecte une dose initiale de 0,2 à 0,5mg, puis réinjections de 0,1-0,2mg jusqu’à obtention de l’effet cardiovasculaire désiré. En cas de collapsus persistant, comme dans notre cas, l’adrénaline est poursuivie en continue à la dose de 5 à 20µg/kg/mn. L’administration de corticoïdes (cortisone 200 mg IV) renouvelée toutes les 6 heures est habituellement proposée dans le cadre de la prévention des manifestations récurrentes de l’anaphylaxie [6,7].

Bien qu’il soit classiquement décrit une évolution par vagues du choc anaphylactique, cela est rarement constaté en anesthésie. Il s’agit plutôt de résistance au traitement ou de rechute lorsque l’on allège le débit des catécholamines. Ces risques imposent une surveillance de 24 heures en soins intensifs, même si les patients présentent une rémission de leur symptomatologie.

## Conclusion

Le choc anaphylactique est une complication connue de la rupture intra péritonéale spontanée ou post-traumatique du kyste hydatique du foie. Sa survenue en peropératoire reste imprévisible et de plus en plus exceptionnelle. Sa prévention est chirurgicale, en évitant la manipulation du kyste, de le vider avant d’injecter le scolicide; et de ne pas l’administrer sous forte pression.
